# Treatment of Autoimmune Inflammation by a TLR7 Ligand Regulating the Innate Immune System

**DOI:** 10.1371/journal.pone.0045860

**Published:** 2012-09-28

**Authors:** Tomoko Hayashi, Shiyin Yao, Brian Crain, Michael Chan, Rommel I. Tawatao, Christine Gray, Linda Vuong, Fitzgerald Lao, Howard B. Cottam, Dennis A. Carson, Maripat Corr

**Affiliations:** 1 Moores Cancer Center, University of California San Diego, La Jolla, California, United States of America; 2 Department of Medicine, University of California San Diego, La Jolla, California, United States of America; National Jewish Health and University of Colorado School of Medicine, United States of America

## Abstract

The Toll-like receptors (TLR) have been advocated as attractive therapeutic targets because TLR signaling plays dual roles in initiating adaptive immune responses and perpetuating inflammation. Paradoxically, repeated stimulation of bone marrow mononuclear cells with a synthetic TLR7 ligand 9-benzyl-8-hydroxy-2-(2-methoxyethoxy) adenine (called 1V136) leads to subsequent TLR hyporesponsiveness. Further studies on the mechanism of action of this pharmacologic agent demonstrated that the TLR7 ligand treatment depressed dendritic cell activation, but did not directly affect T cell function. To verify this mechanism, we utilized experimental allergic encephalitis (EAE) as an *in vivo* T cell dependent autoimmune model. Drug treated SJL/J mice immunized with proteolipid protein (PLP)_139–151_ peptide had attenuated disease severity, reduced accumulation of mononuclear cells in the central nervous system (CNS), and limited demyelination, without any apparent systemic toxicity. Splenic T cells from treated mice produced less cytokines upon antigenic rechallenge. In the spinal cords of 1V136-treated EAE mice, the expression of chemoattractants was also reduced, suggesting innate immune cell hyposensitization in the CNS. Indeed, systemic 1V136 did penetrate the CNS. These experiments indicated that repeated doses of a TLR7 ligand may desensitize dendritic cells in lymphoid organs, leading to diminished T cell responses. This treatment strategy might be a new modality to treat T cell mediated autoimmune diseases.

## Introduction

The innate immune system forms part of the first line defense of barrier tissues and immuno-privileged sites. Toll-like receptors (TLRs) are an integral part of this host innate immune defense. TLR ligands were initially described as conserved molecular signatures of pathogens, but TLRs also interact with endogenous ligands released by necrotic cells and this process could potentially intensify autoimmune diseases such as multiple sclerosis (MS) [1]. The discovery that synthetic molecules can bind specific TLRs has generated interest for the development of novel therapeutics for diseases that involve innate immunity. TLR7 recognizes naturally occurring single strand (ss) RNA and synthetic low molecular weight ligands, including imidazoquinolines, and purine-like molecules [2], [3], [4]. Among the latter, 9-benzyl-8-hydroxy-2-(2-methoxyethoxy) adenine (SM360320; designated here as 1V136), has been shown to be a potent and specific TLR7 agonist [5]. We previously demonstrated that repeated administration of the synthetic TLR7 ligand (1V136) reduced myeloid differentiation primary response gene 88 (MyD88) signaling, and impaired the signaling ability of TLR2, TLR7, or TLR9 activators [6]. The concomitant pharmacological down-regulation of the MyD88 signaling pathway was neuroprotective *in vivo* and attenuated inflammatory responses in the myelin oligodendrocyte glycoprotein (MOG) peptide induced experimental allergic encephalitis (EAE) model of multiple sclerosis (MS) [6]. However the in vivo mechanism of action of the drug was not determined [6].

The reduction of clinical signs in a T cell dependent disease model was surprising given that the drug targeted a receptor of the innate immune system. In the central nervous system (CNS), the immune system is tightly controlled to prevent excessive injury or inflammation, and to promote repair of injured neural tissue. The resident cells within the CNS, i.e. glial cells and neurons, are able to produce a wide range of immune mediators to control local inflammation [7]. Stimulation of infiltrating macrophages, as well as microglia and astrocytes has been implicated in the pathology of MS [8]. However, T and B cells also enter into the CNS of MS patients suggesting that induction of adaptive immune responses may instigate the inflammatory attack [9]. Hence, we further examined 1V136 treatment to dissect the drug’s influence on innate immune cells and T cells.

To investigate the cellular mechanisms of TLR7 hyposensitization we performed a series of *ex-vivo* experiments with ovalbumin (OVA) as a test antigen to examine the ability of 1V136 to render T cells or antigen presenting cells (APCs) refractory to stimulation. *In vitro* 1V136 limited the activation of bone marrow derived dendritic cells (BMDC) and attenuated their ability to stimulate an antigen specific recall response. In mixed cultures with cells from *Tlr7^−/−^* and wild type (WT) T cells the drug did not directly affect T cells. We extended these studies to the EAE model where SJL/J mice immunized with peptide from myelin proteolipid protein (PLP)_139–151_ [10] were treated with daily doses of 1V136 or vehicle after the antigen priming phase beginning on day 6. The drug penetrated the CNS, attenuated disease manifestations, and reduced antigen specific T cell responses. There was a reduction in the cellular infiltrate in 1V136 treated EAE mice, suggesting that 1V136 also influenced the ability of spinal cells to recruit inflammatory cells to the target tissue. These data suggested that reduction in innate immune stimulation can attenuate the damage associated with an adaptive immune response.

## Results

### TLR7 Ligand Treatment Reduced Antigen-specific T Cell Responses

**Figure 1:**
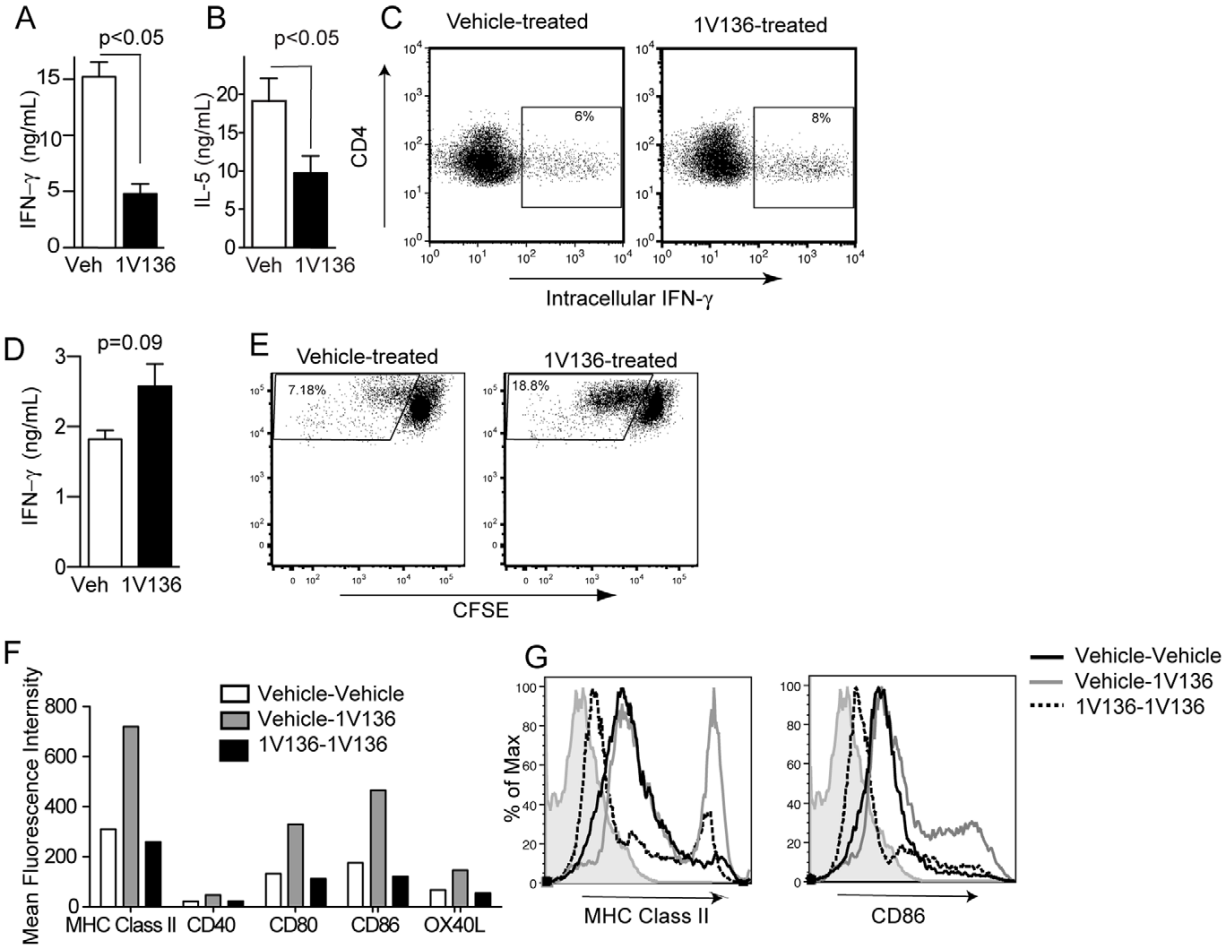
TLR7 ligand treatment reduced antigen specific T cell responses and dendritic cell activation. C57BL/6 mice (n = 5/group) were intradermally immunized with OVA (50 µg) mixed with (A) ISS-ODN (50 µg) or (B) alum (0.5 mg) on days 0 and 7. Immunized mice were treated daily with 1V136 (150 nmol/mouse) (black bar) or vehicle (white bar) s.c. from day 7 onward. On day 21 mice were sacrificed and splenocytes were harvested. Splenocytes were cultured with OVA (100 µg/ml) *ex vivo* for 5 days, and IFN-γ (A) or IL-5 (B) in the culture supernatant was measured by ELISA. p<0.05 by Student t test compared to vehicle treated. (C) On the day of harvest the splenocytes were also nonspecifically stimulated with PMA and ionomycin, and CD4^+^ T cells were analyzed by flow cytometry for intracellular IFN-γ. Data shown are representative of three independent experiments that exhibited similar results. (D) C57BL/6 mice (n = 5/group) were intradermally immunized with OVA (50 µg) mixed with ISS-ODN (50 µg) on days 0 and 7. Immunized mice were treated daily with 1V136 (150 nmol/mouse) (black bar) or vehicle (white bar) s.c. from day 5 onward. On day 21 mice were sacrificed and splenocytes were harvested and enriched for CD4^+^ cells by magnetic beads. The enriched CD4^+^ T cells were cultured with OVA (100 µg/ml) and BMDCs for 3 days and IFN-γ in the culture supernatant was measured by ELISA. p<0.05 by Student t test compared to vehicle treated. (E) An aliquot of the enriched CD4^+^ T cells above were CFSE labeled, and cultured for 3 days with BMDC and OVA. The proliferation was assessed by flow cytometry. (F) BMDCs were treated with 1V136 (1 µM) or vehicle for 18 h, washed and restimulated with 1V136 (1 µM). The expression of MHC class II, CD40, CD80, CD86, or OX40L in the gated CD11c population were evaluated by flow cytometry. The data shown are representative of three independent experiments. (G) Representative histogram plots of MHC class II and CD86 in the CD11c^+^ gated populations are shown. Light gray areas are isotype controls.

As EAE is a T cell driven disease, we examined if treatment with an innate immune stimulating agent (1V136) could influence an antigen specific T helper (Th) cell response after the initial priming phase. Hence, mice were immunized in a Th1 or a Th2 skewing strategy with an immunostimulatory oligonucleotide (ISS-ODN) as a defined Th1 stimulating adjuvant or alum as a Th2 adjuvant, and OVA as a test antigen on days 0 and 7. We previously found that a subcutaneous dose of 1V136 at 150 nmoles daily was well tolerated, suppressed peritoneal inflammation and did not stimulate a systemic proinflammatory response [11]. From day 7 onward the mice were treated with s.c. vehicle or 150 nmoles 1V136 per day. On day 21 the mice were sacrificed and the splenocytes were restimulated with OVA *ex-vivo*. There was a significant reduction in the amount of interferon (IFN)-γ and IL-5, Th1 and Th2 cytokines, respectively, released compared to cells from vehicle treated mice (Figure 1A and 1B, p<0.05). However, splenocytes from the same vehicle and 1V136 treated mice stimulated on the day of harvest with PMA/ionomycin had comparable numbers of cells capable of producing IFN-γ by intracellular cytokine staining (Figure 1C).

To determine if the reduction in the restimulated cytokine response was from the T cells or the splenocyte APCs from the 1V136 treated mice we immunized mice with ISS-ODN and OVA as above. From day 7 onward the mice were treated with s.c. vehicle or 150 nmoles 1V136 per day. On day 21 the mice were sacrificed and the spleens were harvested. The CD4^+^ T cells were enriched with magnetic beads and then restimulated in a co-culture with freshly prepared bone marrow derived dendritic cells (BMDC) in the presence of OVA. The CD4^+^ T cells from the 1V136 treated mice were capable of releasing equivalent amounts of IFN-γ as the vehicle treated cells (p = 0.09, Figure 1D). This result indicated that the APCs in Figure 1A and 1B were not capable of stimulating the T cells maximally, whereas the freshly prepared BMDC could elicit the recall response of primed T cells. In addition, the enriched CD4^+^ T cells were labeled with **c**arboxyfluorescein succinimidyl ester **(**CFSE) and cultured with BMDC and OVA for three days. The CD4^+^ T cells from 1V136 treated mice had a greater *in vitro* proliferative response (Figure 1E). It is unlikely that CD4^+^ regulatory T cells were responsible for the reduced response in Figure 1A as they should still be present in these cultures and the response of T cells from 1V136 treated mice is not reduced with fresh APCs.

In parallel the effect of 1V136 desensitization on dendritic cells was tested. Wild type (WT) BMDCs were pretreated with 1V136 or vehicle overnight and then challenged with 1V136 to activate TLR7 and test desensitization. The vehicle pretreated cells rapidly upregulated their surface costimulatory molecules in response to 1V136 challenge (vehicle-1V136). However, the 1V136 pretreated BMDCs did not augment their expression of MHC class II, and costimulatory molecules: CD80, CD86, and OX40 ligand (L) by FACS analysis after TLR7 ligand challenge (Figure 1F and 1G). Pretreatment of 1V136 desensitized the ability of the BMDCs to upregulate their costimulatory molecules in an activation response to a TLR7 stimulation challenge (1V136-1V136). Taken together these data suggested a mechanism that 1V136 acted via DCs, rather than by a direct effect on the T cells.

### TLR7 Ligand Treatment Targets Antigen Presenting Cells, not T Cells

**Figure 2:**
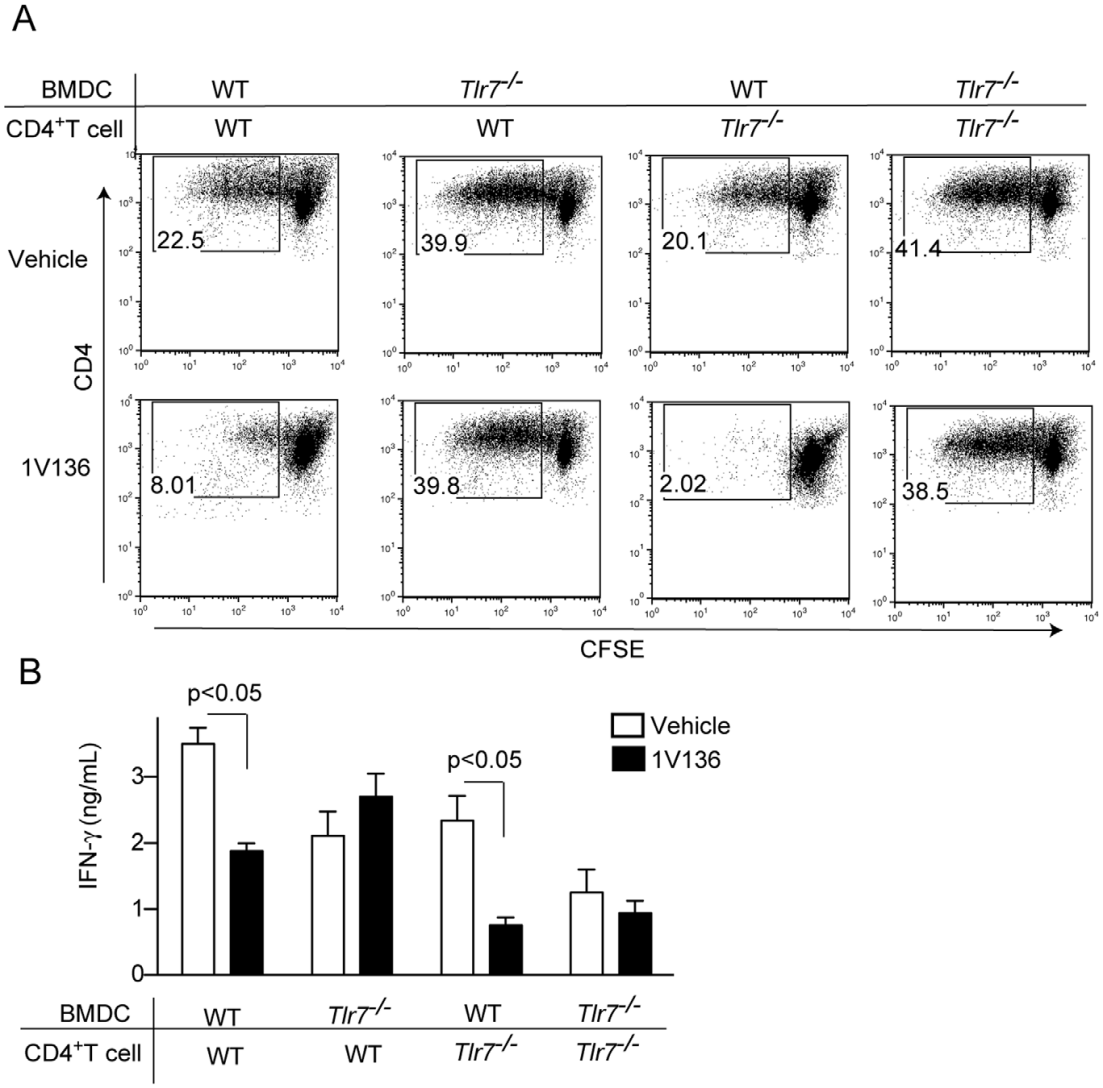
TLR7 ligand treatment selectively impaired dendritic cell and not T cell function. (A) CFSE-labeled CD4^+^ T cells were prepared from WT or *Tlr7^−/−^* mice immunized with OVA/CFA. WT or *Tlr7^−/−^* BMDCs were incubated with these primed CD4^+^ T cells with OVA (100 µg/mL) in the presence or the absence of 1V136 (1 µM) for five days. T cell proliferation was monitored by flow cytometry. Representative plots are shown from three experiments. (B) The culture supernatants were assessed for IFN-γ levels by ELISA. The data shown are representative of two independent experiments. p<0.05 by Student *t* test compared to vehicle control.

To distinguish whether the 1V136 treatment directly affected T cells or BMDCs, or both, we performed co-culture experiments. Preprimed CD4^+^ T cells from WT or TLR7 deficient mice that had been immunized with Complete Freunds Adjuvant (CFA) and OVA, were co-cultured with WT or TLR deficient BMDCs with OVA, and 1V136 (or vehicle). Cultures with TLR7 deficient BMDCs were refractory to desensitization by 1V136 and stimulated T cell proliferation despite the presence of 1V136 (Figure 2A). However, the WT BMDCs did not support T cell proliferation when treated with 1V136 (Figure 2A). T cell proliferation and antigen-specific IFN-γ production were not affected by the TLR7 status of the T cells (Figures 2A and 2B). Hence, the 1V136, TLR7 ligand, treatment was only effective when TLR7 was expressed in the APCs.

### Low Dose TLR7 Ligand Treatment Mitigates the Inflammation in EAE

**Figure 3:**
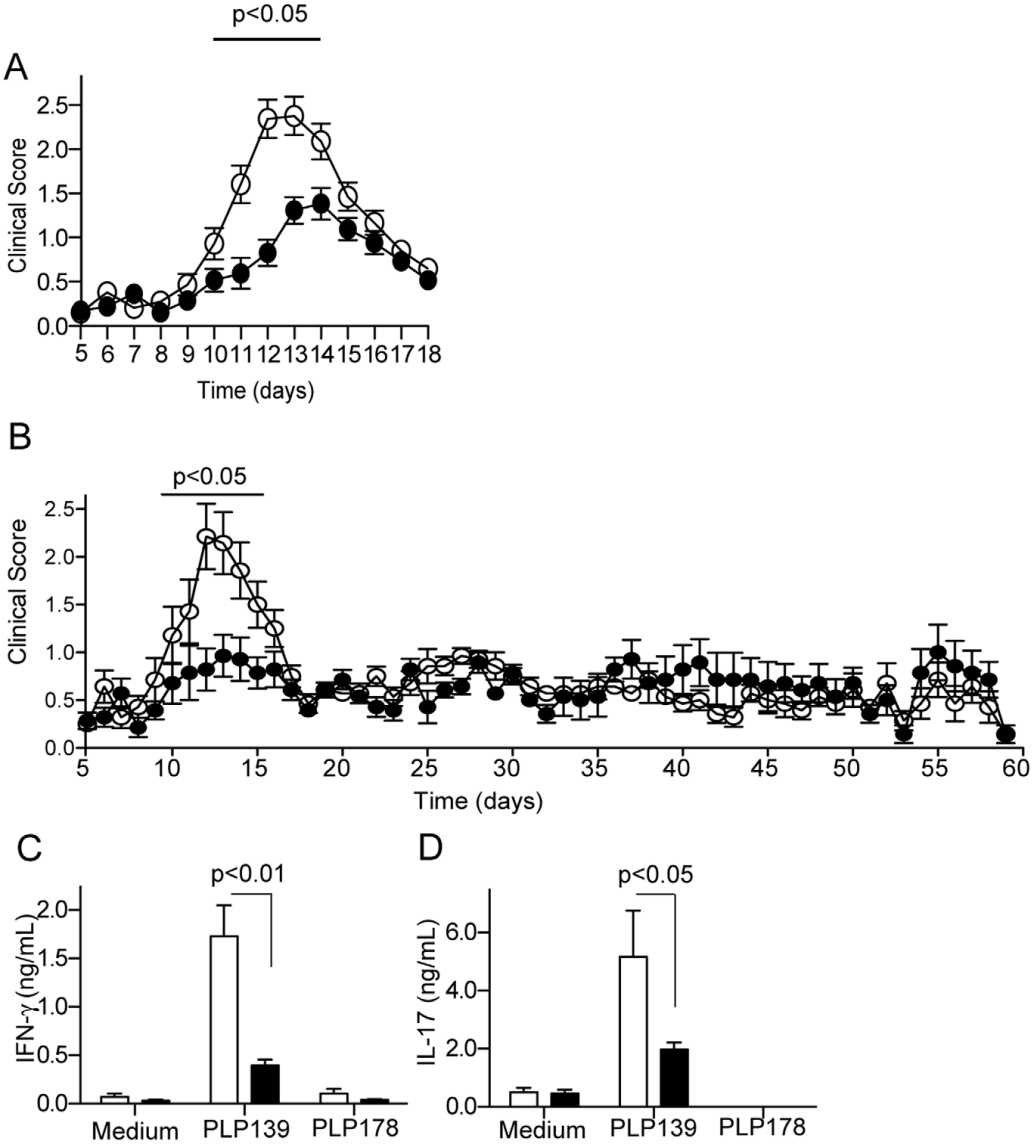
TLR7 ligand treatment reduced clinical severity, and T cell responses of EAE mice. (A) SJL/J mice were i.p. immunized with PLP_139–151_ in CFA with Ptx. Mice (n = 26/group) received 150 nmol/animal 1V136 or vehicle s.c. daily from day 6 onward. The mice were scored for disease and the data were pooled from four independent experiments. Bar indicates *p*<0.05 by two-way ANOVA with Bonferroni *post hoc* tests. (B) SJL mice were immunized as above (n = 14/group) and treated from day 5 to 18 with 150 nmol/animal 1V136 or vehicle s.c. and disease was monitored up to 60 days. Data are pooled from 2 independent experiments and the bar indicates *p*<0.05 by two-way ANOVA with Bonferroni *post hoc* tests. (C, D) Splenocytes obtained from vehicle- (n = 5) and 1V136-treated (n = 5) SJL/J EAE mice at 19 days were restimulated *in vitro* with PLP_139–151_ peptide for 3 days. Levels of IFN-γ (C), and IL-17 (D) in the supernatants were determined by ELISA. Data shown are means ± SEM and are representative of three independent experiments that showed similar trends. p<0.05 compared vehicle-treated group by Student t test.

Previously we reported that repeated low dose administration of a TLR7 ligand limited the course of neural inflammation in the MOG model of EAE in C57BL/6 mice [6]. To validate the efficacy, and to determine the mechanism of low dose TLR7 agonism in another mouse model of MS, we used PLP_139–151_ peptide induced EAE in SJL/J mice. By using a different peptide, and a strain with a different major histocompatilbility complex (MHC) haplotype, we would also be able to assess the robustness of the 1V136 hyposensitizing strategy. In addition the effect of treatment could be tested in preventing relapse. SJL/J mice were immunized with PLP_139–151_ peptide in CFA and given *Bordetella pertussis* toxin on days 0 and 2. Daily treatment with 150 nmole s.c. 1V136 in PLP_139–151_-immunized mice was initiated on day 6 (before the mice had visible clinical symptoms), and completed on day 18 (when symptoms in vehicle-treated mice had lessened) (Figure 3A). Mice treated daily with the TLR7 ligand exhibited significantly lower clinical scores from day 12 to 16 compared to the vehicle-treated group (p<0.05, Figure 3A). In extended experiments, the mice were treated from day 5 to 18 as above but were monitored up to 60 days. There was no effect of the treatment on subsequent relapse (Figure 3B).

### Hyposensitization with 1V136 is Accompanied by Reduced Peripheral Th1 and Th17 Responses

EAE has been reported to rely heavily on Th1 and Th17 responses [12]. Hence, we evaluated whether TLR7 ligand treatment affected antigen specific Th1 or Th17 immune responses to two different PLP peptide epitopes. Treatment with 1V136 significantly reduced PLP_139–151_ specific IFN-γ and IL-17 secretion by splenocytes (Figure 3C and 3D). There were no significant responses to the control peptide PLP_178–191_. In addition, the total numbers of T cells, B cells and myeloid cells in the spleens were not affected by 1V136 (Figure S1). Despite the reduction in IFN-γ and IL-17 release in splenocyte cultures restimulated with antigen, there was no significant difference in the serum levels of IgG1 and IgG2c specific for PLP_139–151_ between vehicle- and 1V136-treated mice (Figure S2).

### Hyposensitization with 1V136 Results in Reduced Cellular Influx and Demyelination

**Figure 4:**
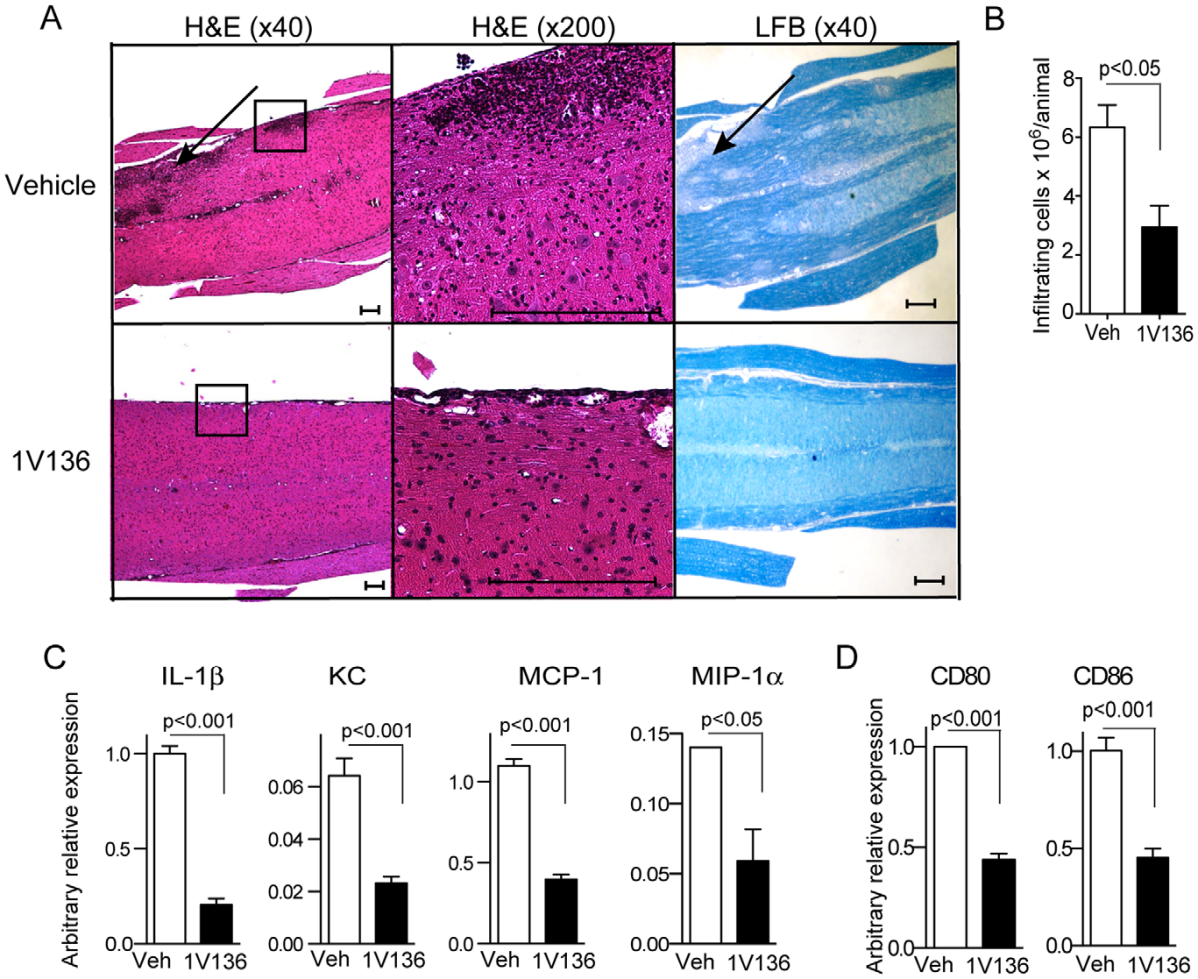
TLR7 ligand treatment reduced spinal cellular infiltration, and spinal chemokines and cytokines of EAE mice. (A) Representative sagittal sections of lumbar spinal cords from vehicle-treated or 1V136-treated EAE mice. H&E stained sections are shown in the left panels (×40 original magnification) and the insets are shown at higher magnification (middle panels, ×200 original magnification). Areas of demyelination are indicated by the arrows in the LFB stained sections (right panels). Scale bars:100 µm. (B) The total number of infiltrating cells isolated from pooled spinal cords from vehicle-treated (Veh) or 1V136-treated (1V136) EAE mice (n = 5/group). The data shown are averages of three independent experiments ± SEM. *p*<0.05 by Student t test. (C) 1V136 treatment reduced chemokine expression in the spinal cords in the EAE mice. Chemokine expression levels in the spinals cords were reduced in 1V136-treated EAE mice. RNA was extracted from pooled spinal cord samples collected from 1V136-treated (n = 4) or vehicle-treated (n = 4) mice. mRNA levels of IL-1β, KC, MCP-1 or MIP-1α were analyzed by qPCR methods. The data were normalized to 18S ribosomal RNA gene and are presented as arbitrary units. *p*<0.05 by Student t test. Data shown are representative of two independent experiments that exhibited similar results. (D) The levels of CD80 and CD86 mRNA transcripts were also reduced in the spinal cords of 1V136 treated mice. Data are presented as arbitrary units as normalized to GAPDH. *p*<0.05 by Student *t* test.

Demyelination is a significant pathologic feature of MS and contributes to functional impairment [13], [14]. Inflammatory cell infiltration in the white matter of spinal cords results in tissue damage and demyelination [14]. We therefore evaluated the lumbar spinal cords on day 19 for inflammatory cell infiltration by histology (Figure 4A). In the vehicle-treated mice, there were prominent areas of leukocyte infiltration in the spinal cord (Figure 4A) as indicated by the arrow and inset. Serial sections of lumbar spinal cords from vehicle-treated EAE mice revealed that demyelination generally correlated with areas of cell infiltration (black arrow in right panels, Figure 4A). By contrast, leukocyte infiltration and demyelination were minimal in 1V136-treated mice (lower panels in Figure 4A).

To characterize the infiltrating cells in the spinal cords of vehicle and 1V136-treated mice the entire spinal cords were enzymatically digested. The released immune cells were enriched by Percoll gradient centrifugation. The 1V136 treatment significantly reduced the total number of infiltrating cells in the spinal cords (Figure 4B). These cells were stained for CD4 and CD8, B220, CD11b or Gr1 to identify T cells, B cells and myeloid cells by flow cytometry (Figure S3). In the 1V136-treated animals, there was an overall decrease in the number of infiltrating cells, and a marked reduction in CD4^+^ T cells (23.5% vs. 11.9% in vehicle vs. 1V136-treated, respectively) compared to CD8^+^ T cells (3.55% vs. 3.17%). The infiltration of B220^+^ cells was also reduced (8.59% vs. 1.84%). Although the relative percentage of CD11b^+^Gr1^+^ cells increased (0.75% vs. 0.9%) after 1V136 treatment, the total number of CD11b^+^ Gr1^+^ cells was reduced (3.3×10^6^ vs. 1.7×10^6^) (Figure S3). Representative serial sections for immunohistochemistry also showed a reduction in F4/80^+^, CD3^+^ and B220^+^ cells in the spinal cords of 1V136 treated mice from Day 19 (Figure S4).

### TLR7 Ligand Treatment Reduced the Levels of Chemoattractants in EAE Spinal Cords

Trafficking of immune cells to target organs or tissues depends on numerous factors, such as chemotactic cytokines and chemokines at inflammatory sites [15]. Hence, we studied whether chemokine expression in spinal cords was influenced by TLR7 ligand treatment. Spinal cords from 1V136-treated EAE mice showed reduced levels of mRNA transcripts of cytokines and chemokines that recruit inflammatory cells including: IL-1β, KC, MCP-1 and MIP-1α, compared to those of vehicle-treated EAE mice (Figure 4C). In contrast to the spinal cords, only the IL-1β level was reduced in the sera on day 19 after resolution of the clinical signs (Figure S5). The significant reduction in CD80 and CD86 mRNA in the spinal cords of 1V136 treated mice suggests a reduction in activation or number of infiltrating APCs (Figure 4D).

### 1V136 Penetrates the CNS and Attenuates the Activation of Microglia

**Figure 5:**
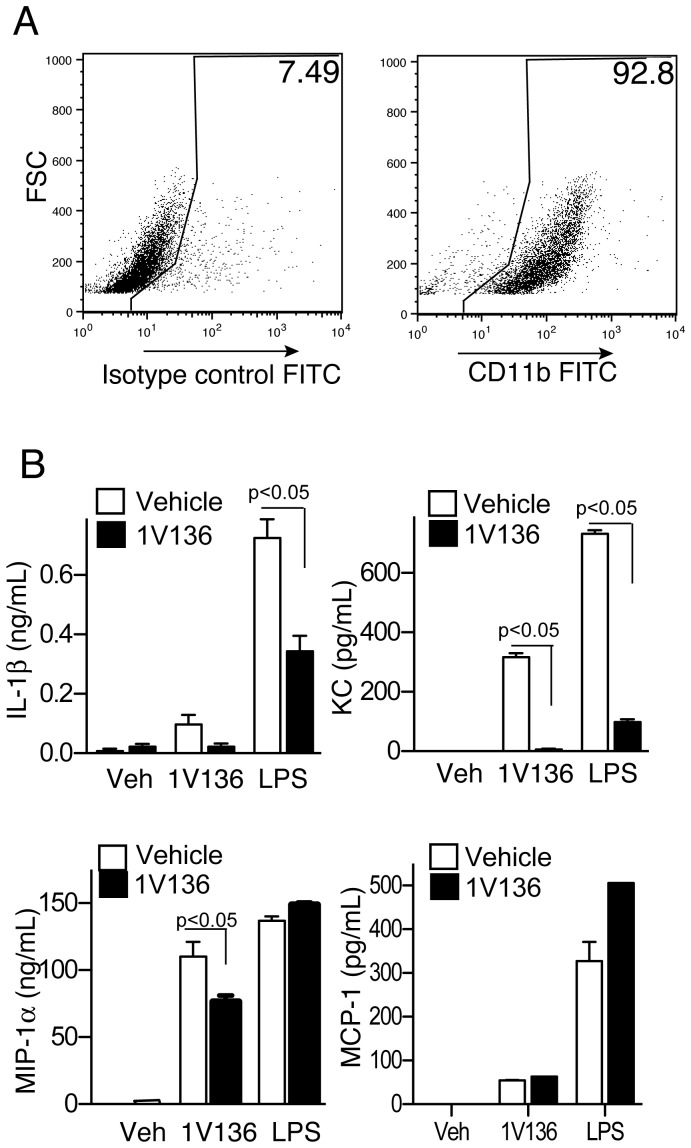
TLR7 ligand treatment induced hyporesponsiveness in primary CNS CD11b-enriched cultures. (A) Mixed glial cells were cultured from the brains neonatal mice and tested for enrichment after 6 weeks by flow cytometry using anti-CD11b antibodies. (B) These primary microglial cultures were treated with vehicle (white bar) or 1 µM 1V136 (black bar) overnight. The cells were then re-stimulated with vehicle (Veh), 1V136 or LPS for 18 h and supernatants were collected. A panel of cytokines and chemokines was analyzed by Luminex microbead assay for IL-1β, KC, MIP-1α, and MCP-1. Data shown are mean ± SEM of triplicates and are representative of three independent experiments. p<0.05 by Student t test compared to vehicle pretreated cells.

Microglia function as resident antigen presenting cells in the CNS, and express functional TLR7 [16], [17], [18]. Hence, if 1V136 penetrated the blood brain barrier, it might alter microglial function. Ten and 30 minutes after systemic administration we detected 1V136 by liquid chromatography-mass spectrometry (LC-MS) in the brains of perfused WT mice. The brain to plasma ratios of 1V136 ten and 30 min after i.v. drug administration were 0.051 and 0.037, respectively. Hence, we examined the direct effects of 1V136 on primary cells from neonatal brain tissue. Using a protocol to enrich and culture primary microglia, we obtained >90% CD11b^+^ cells (Figure 5A). The CD11b^+^ enriched cells were either pretreated with 1V136 or vehicle for 18 h, and were then restimulated with 1V136, LPS or vehicle on the following day. 1V136-treated microglia secreted less IL-1β, KC, or MIP-1α after LPS or 1V136 re-stimulation (Figure 5B). There was no difference seen in MCP-1 secretion.

## Discussion

In MS patients, the inflammation triggered by effector T cells specific to CNS antigens is responsible for the pathological features of the disease, which include demyelination, oligodendrocyte loss and axonal injury [13]. The innate immune response might also contribute to MS disease progression [19] by promoting proinflammatory cytokine release and/or by regulating the function of APC [20]. In murine models of EAE the innate immune system has been shown to play a distinct role in initiating and sustaining the severity of disease [21], [22], [23]. MyD88 null mice were completely EAE resistant and this protection was partly due to engagement of TLR4 or TLR9 [21], [22]. By contrast, activation of the MyD88 independent TLR pathway mitigated disease [23].

In a previous report, we demonstrated the beneficial effects of chronic administration of a TLR7 ligand on the clinical manifestations of EAE in MOG-immunized C57BL/6 mice (H-2^b^) [6]. In the current study, we used PLP_139–151_-immunized SJL/J (H-2^s^) mice to dissect the mechanisms of the drug’s action. The anti-inflammatory effect of the TLR7 agonist was independent of MHC haplotype (H-2^b^ vs. H-2^s^) and the specific peptide utilized to induce autoimmune CNS disease. Rather, the TLR7 ligand treatment reduced immune cell infiltration in the spinal cord, and T cell mediated responses via a direct effect on APC in the periphery, and possibly in the central nervous system. The effect of the treatment was limited to the time when the drug was administered.

TLR7 stimulation can influence T cell function directly or indirectly. In humans, TLR7 is most abundantly found in plasmacytoid DCs, although myeloid DCs, tissue mast cells, epithelial cells and possibly other cell types can variably express this TLR [24], [25], [26], [27]. In mice, TLR7 expression by microglial cells has been clearly demonstrated [28], [29]. Also recent reports indicated that some CD4^+^ T cells express TLR7 [25], [30]. Thus, systemic TLR7 ligand induced reduction in antigen specific T cell responses could be attributable to drug effects on the APCs, T cells, or both cell types. Splenocytes harvested from EAE mice treated with 1V136 secreted significantly less IFN-γ and IL-17 than those from vehicle-treated EAE mice after *in vitro* restimulation with antigens. The reduced cytokine secretion was not due to reduced numbers of splenic T cells, nor dendritic cells (Figure S1). Mixed cultures with TLR7-sufficient and TLR7-null cells indicated that the hyposensitization occurred when TLR7 was expressed by the BMDCs, whereas the receptor was not required on the T cell (Figure 2).

In this EAE model, TLR7 ligand treatment reduced leukocyte infiltration specific to the site of inflammation, the spinal cord. The diminished cellular infiltrate was concordant with lower expression levels of multiple chemokines in the spinal cords of 1V136-treated mice compared to these of vehicle-treated mice. In contrast to the spinal cord, the serum levels of most of cytokines and chemokines (except IL-1β ), were unchanged by 1V136 treatment (Figure S4). However, the serum levels were assessed at the end of the clinical course and not at the peak of disease, and there might have been a difference earlier in the course of the illness. These data lead us to hypothesize that the immunomodulatory TLR7 ligand treatment might have reduced chemoattractant release at the site of inflammation in the spinal cord. In EAE, a likely source for these chemokines is the resident non-neuronal microglial cells.

As 1V136 is a small molecule, it was feasible that this compound would be able to penetrate the blood brain barrier [5]. We were able to detect the drug in the brain tissue of mice that had been injected i.v. with 1V136. The presence of 1V136 in the CNS suggested that 1V136 might also be influencing inflammation at this site. In the mouse, microglia, astrocytes and oligodendrocytes express TLRs and function as innate immune cells in the CNS [18], [28]. Our data showed that 1V136 could induce hyporesponsiveness in primary cultures enriched for microglia (Figure 5). However there are other RNA sensors such as RIG-1 like helicases that are active in the innate immune response and can also modulate EAE [31].

In a healthy individual, there is a balance of proinflammatory and anti-inflammatory cascades so that immune surveillance is maintained without continuous inflammation. As shown here, TLR7 ligand treatment could mitigate T cell mediated autoimmune disease through desensitization of sentinel innate immune cells including antigen presenting DC as well as microglia. Ancillary mechanisms and cascading events, such as interferon receptor modulation or ligation, also warrant further investigation [31], [32]. Modulation of the immune system by this TLR7 ligand treatment is reversible without inducing long term immunoparalysis that could increase susceptibility to infection [6], [33]. In addition 1V136 is orally bioavailable, and could potentially be combined with other treatments [5], [34]. The Food and Drug Administration (FDA) has approved topical TLR7 active therapies and experimental systemic treatments have been well tolerated in humans [35], [36], [37], [38], [39], [40], [41].

## Materials and Methods

### Animals

SJL/J and C57BL/6 mice were purchased from The Jackson Laboratories (Bar Harbor, MA) and Charles River Laboratories (Wilmington, MA), respectively. TLR7 deficient mice were a gift from Dr. S. Akira (Osaka University, Osaka, Japan) and bred onto the C57BL/6 background at University of California, San Diego (UCSD). This study was carried out in strict accordance with the recommendations in the Guide for the Care and Use of Laboratory Animals of the National Institutes of Health. The University of California San Diego Institutional Animal Care and Use Committee approved these experiments (Protocol Number: S00028).

### Reagents

PLP_139–151_ (HSLGKWLGHPDKF) and PLP_178–191_ (NTWTTCQSIAFPSK) peptides were synthesized by Genemed Synthesis (San Antonio, TX). Complete Freund’s Adjuvant (CFA) containing 0.4 mg of *Mycobacterium tuberculosis* H37Ra and IFA were obtained from Chondrex (Redmond, WA) and *Bordetella pertussis* toxin (Ptx) was obtained from List Biological (Campbell, CA). LPS (*Escherichia coli* 026:B6) was purchased from Sigma Chemical Co. (St Louis, LA). HBSS, RPMI 1640 medium (Invitrogen, Carlsbad, CA), DMEM (Invitrogen) were supplemented with 10% FBS (Sigma) and penicillin/streptomycin (Sigma). Immunostimulatory sequence oligodeoxynucleotides (ISS-ODN) was synthesized by Trilink Biotechnologies (San Diego CA) [42]. OVA, collagenase I and DNase I were obtained from Worthington Biochemical Corporation (Lakewood, NJ). TLR7 ligand, 1V136, was synthesized in our laboratory as previously described [43]. 1V136 was dissolved in DMSO as a 100 mM stock solution and kept at −20°C until use. The stock solution was diluted in normal saline to a final volume of 100 µL per dose with a final DMSO concentration of 0.5%, which was also used as the vehicle control. Endotoxin levels in all reagents were measured using the QCL1000 end point chromogenic Limulus Amoebocyte Lysate assay purchased from Lonza (Walkerville, MD).

### Bone Marrow Dendritic Cell Cultures

BMDCs from C57BL/6, or *Tlr7^−/−^* mice (C57BL/6 background) were prepared as described previously [44]. BMDCs were stained with antibodies against CD11b, CD11c, CD40, CD80, CD86, MHC class II (I-A/I-E) or OX40L (eBioscience). Flow cytometry was performed using a FACSCalibur cytometer (BD Bioscience) and the data were analyzed using FlowJo software (Ashland, OR).

### Immunization with OVA and T Cell Responses

C57BL/6 (WT) mice were immunized with OVA (100 µg/mouse), and alum (0.5 mg, Brenntag Biosector A/S, Denmark), ISS-ODN (50 µg/mouse) or CFA on days 0 and 7. The mice were daily treated with 150 nmol 1V136 from day 7 onward and were sacrificed day 21. To evaluate the frequency of IFN-γ producing CD4^+^ cells, splenocytes were cultured with PMA (50 ng/ml, Sigma) and ionomycin (1 µg/ml, Sigma) for four hours in the presence of Golgistop (BD Bioscience). Fixed and permeabilized cells were stained with anti-IFN- γ antibody and detected by flow cytometry (according to manufacturer’s instructions; BD Bioscience). Additional splenocytes (200 µl of 5×10^6^/ml of a 96 well plate) were cultured in triplicate wells with 100 µg/mL OVA for cytokine release. IFN-γ and IL-5 in the supernatant was measured by ELISA (paired antibodies from BD Bioscience). In other experiments, C57BL/6 or *Tlr7^−/−^* mice were immunized with OVA (50 µg) mixed with CFA on days 0 and 7. OVA-primed CD4^+^ T cells were isolated from immunized mice using an EasySep® Mouse CD4 Positive Selection Kit (Stemcell Technologies, Vancouver, Canada) according to the manufacturer’s instructions. CD4^+^ T cells were labeled with CFSE (Life Sciences) and incubated with BMDCs in the presence or absence of 1 µM 1V136 for 3 or 5 days with OVA. Cell divisions were assessed by flow cytometry and IFN-γ release in the supernatant was measured by ELISA (BD Bioscience).

### Induction, Treatment and Scoring of EAE

Six to eight week old female SJL/J mice were immunized s.c. with 200 µg (PLP)_139–151_ peptide emulsified in CFA in the rear flank. The mice also received 325 ng of Ptx i.p. immediately after immunization and again on day 2. Immunized mice received vehicle or 150 nmol/mouse 1V136 s.c. daily from day 6 to day 18. Mice were monitored daily for clinical signs of EAE from day 5 onward. Clinical scoring included: a score of 1, limp tail; 2, paresis or partial paralysis of the hind limbs; 3, total hind limb paralysis; 4, hind and front limb paralysis; and 5, death. Mice were sacrificed on day 19 or day 60. Spinal cords, sera, and spleens were harvested for subsequent analysis. Splenocytes were cultured in triplicate wells with 10 µg/ml PLP_139–151_, or PLP_178–191_, in RPMI-1640 with 10% FBS (Sigma) and penicillin/streptomycin (Sigma) for 3 days. The levels of IL-17 and IFN-γ in the culture supernatant were determined by ELISA (BD Bioscience). Peptide-specific antibody levels in the sera were measured as previously described [43], [45].

### Histological Analysis

At the time of sacrifice, mice were transcardially perfused with normal saline. The spines were harvested and decalcified with Cal-ExII Fixative/Decalcifier (Fisher Chemical, Houston, TX). The spinal cords were then removed, fixed and embedded in paraffin. Ten µm sections were cut and stained with hematoxylin and eosin (H&E) and Luxol Fast Blue (LFB) to detect myelin. Additional sections were immunostained for F4/80 (AbD Serotec, Oxford, UK), CD3 (BD Bioscience), and B220 (BD Bioscience) by the Histology and Immunohistochemistry Shared Resources at Moores UCSD Cancer Center.

### Isolation of Spinal Cord Infiltrating Cells

The spinal cords were harvested on day 19 and pooled by each experimental group. Spinal cords were minced and incubated in HBSS containing 1 mg/mL collagenase I and 20 µg/mL DNase I for 1 h at 37°C. Infiltrating cells were isolated from the resulting homogenates using a discontinuous 30/70% Percoll Plus gradient (GE Healthcare, Waukesha, WI) [45]. Single cell suspensions from spinal cords, or spleens were stained with antibodies to CD4, CD8, CD25, B220, CD11c, CD11b, or Gr1 (eBioscience) and analyzed by flow cytometry.

### CD11b Enriched Primary Glial Cell Cultures

Microglia enriched populations were prepared from brains of newborn mice (day 0 to 2), which were minced, and digested in Hank’s balanced buffer containing collagenase I (1 mg/mL) and DNase I (20 µg/mL) for 1 h. The cell suspension was filtered through a 100 µm cell strainer. Cells were cultured in a flask coated with poly-L-lysine overnight in DMEM with supplements. Non-adherent cells were removed and further cultured for 12 days. This mixed glial culture was shaken at 200 rpm for 60 minutes at room temperature and the microglia-enriched population was collected (>90% CD11b^+^ by FACS). 5 to 7×10^3^ cells per well in a 96-well plate were incubated with vehicle, or 1 µM 1V136 for 24 hours and then restimulated with 10 µM 1V136 or 0.1 µg LPS overnight. The levels of cytokines and chemokines in the supernatants were studied by Luminex microbead assay (Invitrogen).

### Quantitative Reverse Transcriptase Polymerase Chain Reaction Assay

Total RNA was extracted from whole EAE spinal cords on day 19. Spinal cords were expelled from the spines and were immediately frozen in liquid nitrogen and stored at −80°C. Total RNA isolation, cDNA synthesis, and real time PCR were performed as described previously [6]. The expression levels of CD80 and CD86 were evaluated using Taqman Gene Expression Assay® (Life Technologies). The comparative ^ΔΔ^Ct method was used to measure fold changes in expression of RNA transcript levels between vehicle and drug-treated mice. ^Δ^Ct values were determined by subtracting the average 18 s ribosomal RNA or GAPDH Ct values from each test Ct value. ^ΔΔ^Ct values were normalized by calibrator ^Δ^Ct value. Primers used for the qPCR analysis are listed in Table S1.

### CNS Levels of 1V136

C57BL/6 mice were administered 1 µmol 1V136 in 100 µL saline i.v. and serum was collected 10 and 30 min after the injection. Mice were transcardially perfused with normal saline for 5 min, and brains and spinal cords were harvested. The compound was extracted and area-under-curve (AUC) was measured as described previously [46]. Penetration of 1V136 into the brain parenchyma was estimated by AUC ratio of brain to plasma (B/P ratio) [47].

### Statistical Analysis

Prism 4 (GraphPad Software, San Diego, CA) statistical software was used to establish p-values for comparison between groups of mice (p<0.05 was considered significant). The data are represented as mean ± standard error of the mean (SEM). The Student t test was used to compare two groups and two-way ANOVA was used for multiple group comparisons.

## Supporting Information

Figure S1 **TLR7 ligand treatment does not alter splenocytes cellularity in PLP/EAE mice.** (A) Number of total cell from spleens obtained from vehicle- or 1V136- treated EAE mice. (B) Fluorescent flow cytometric assay of splenocytes. On day 19, splenocytes were harvested and stained for CD4, CD8, B220, Gr1, and CD11b.
(TIF)

Figure S2 **TLR7 ligand treatment does not change myelin specific serum IgG levels in PLP/EAE mice.** On day 19, serum were collected from EAE mice daily treated with vehicle or 1V136. IgG2c (A and B) and IgG1(C and D) specific to PLP_139–151_ (A and C) or PLP_178–191_ (B and D) were measured by ELISA. Data shown are mean ± SEM of representative of three independent experiments.
(TIF)

Figure S3 **Representative FACS plots of spinal cellular infiltrates.** Spinal cellular infiltrates were isolated from vehicle- (upper panels) or 1V136-treated (lower panels) EAE mice on day 19 were stained for CD4, CD8, B220, CD11b or Gr1. Data shown are representative of three independent experiments showing the similar results.
(TIF)

Figure S4 **Representative inmmunohistochemistry stains of lumber spinal cords from vehicle-treated or 1V136-treated EAE mice from** **Figure 4** **.** The sections were stained H&E (a and e) and immunostained for macrophages (F4/80, b and f), B cells (B220, c and g), and T cells (CD3, d and f). × 100 original magnification. Bar indicates 200 µm.
(TIF)

Figure S5 **Serum cytokine profiles of PLP/EAE mice that received TLR7 ligand treatment. Sera were collected from EAE mice treated daily with vehicle (Veh) or 1V136.** Levels of cytokines and chemokines were measured by Luminex beads assay. (A) KC, (B) IP-10, (C) MCP-1,(D) IFN-γ, (E) IL-12, (F) IL-1β, (G) TNFα, and (H) IL-17 levels are means ± SEM and are representative of three independent experiments. *p*<0.05 compared to vehicle-treated mice by Student *t* test.
(TIF)

Table S1 **Primers and probes used in this study.** The sequences for the primer sets and the reference numbers for the prevalidated probes used in this study are listed. The primers were purchased from Integrated DNA Technologies, Inc. (Commercial Park. Coralville, IA) and the probes were from the Universal Probe Library (Roche Diagnostics Corporation, Indianapolis, IN).
(DOCX)
