# Supplementary figures and images for: Treatment of Autoimmune Inflammation by a TLR7 Ligand Regulating the Innate Immune System
Source: PLoS One. 2012 Sep 28;7(9):e45860. doi: 10.1371/journal.pone.0045860 (PMC3461028; doi:10.1371/journal.pone.0045860)

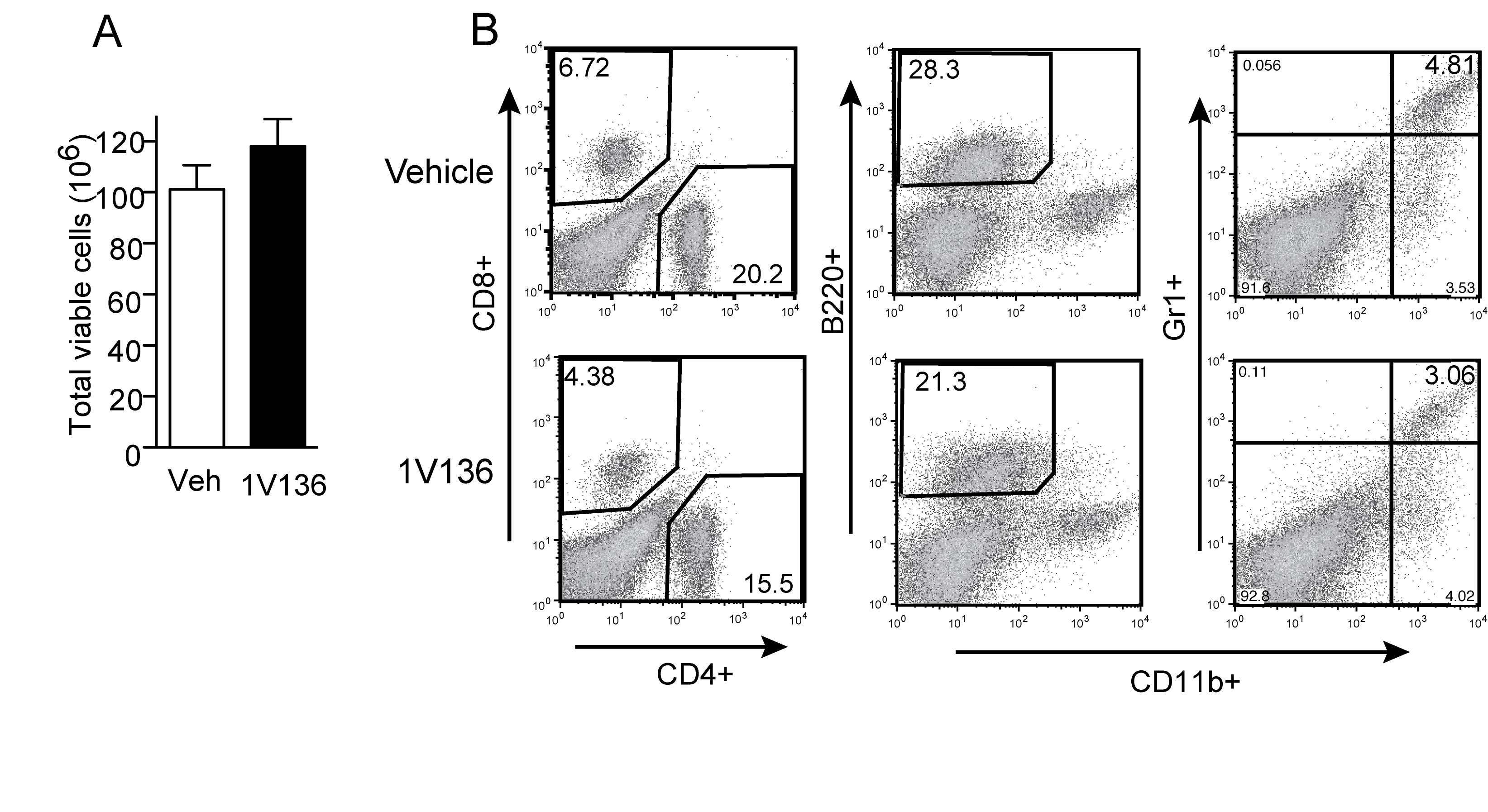

Supplement: Figure S1 — TLR7 ligand treatment does not alter splenocytes cellularity in PLP/EAE mice. (A) Number of total cell from spleens obtained from vehicle- or 1V136- treated EAE mice. (B) Fluorescent flow cytometric assay of splenocytes. On day 19, splenocytes were harvested and stained for CD4, CD8, B220, Gr1, and CD11b. (TIF) [file pone.0045860.s001.tif]

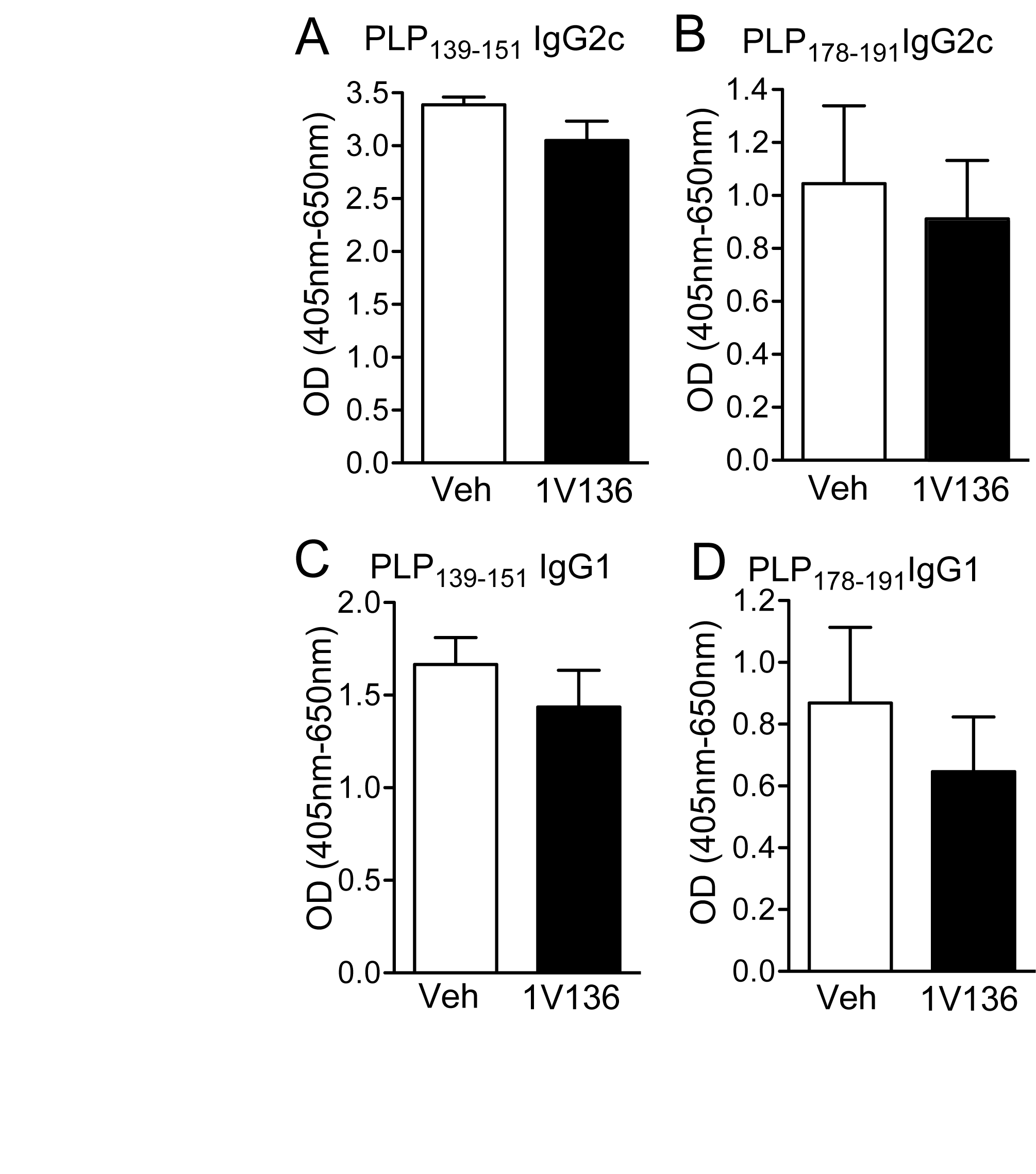

Supplement: Figure S2 — TLR7 ligand treatment does not change myelin specific serum IgG levels in PLP/EAE mice. On day 19, serum were collected from EAE mice daily treated with vehicle or 1V136. IgG2c (A and B) and IgG1(C and D) specific to PLP139–151 (A and C) or PLP178–191 (B and D) were measured by ELISA. Data shown are mean ± SEM of representative of three independent experiments. (TIF) [file pone.0045860.s002.tif]

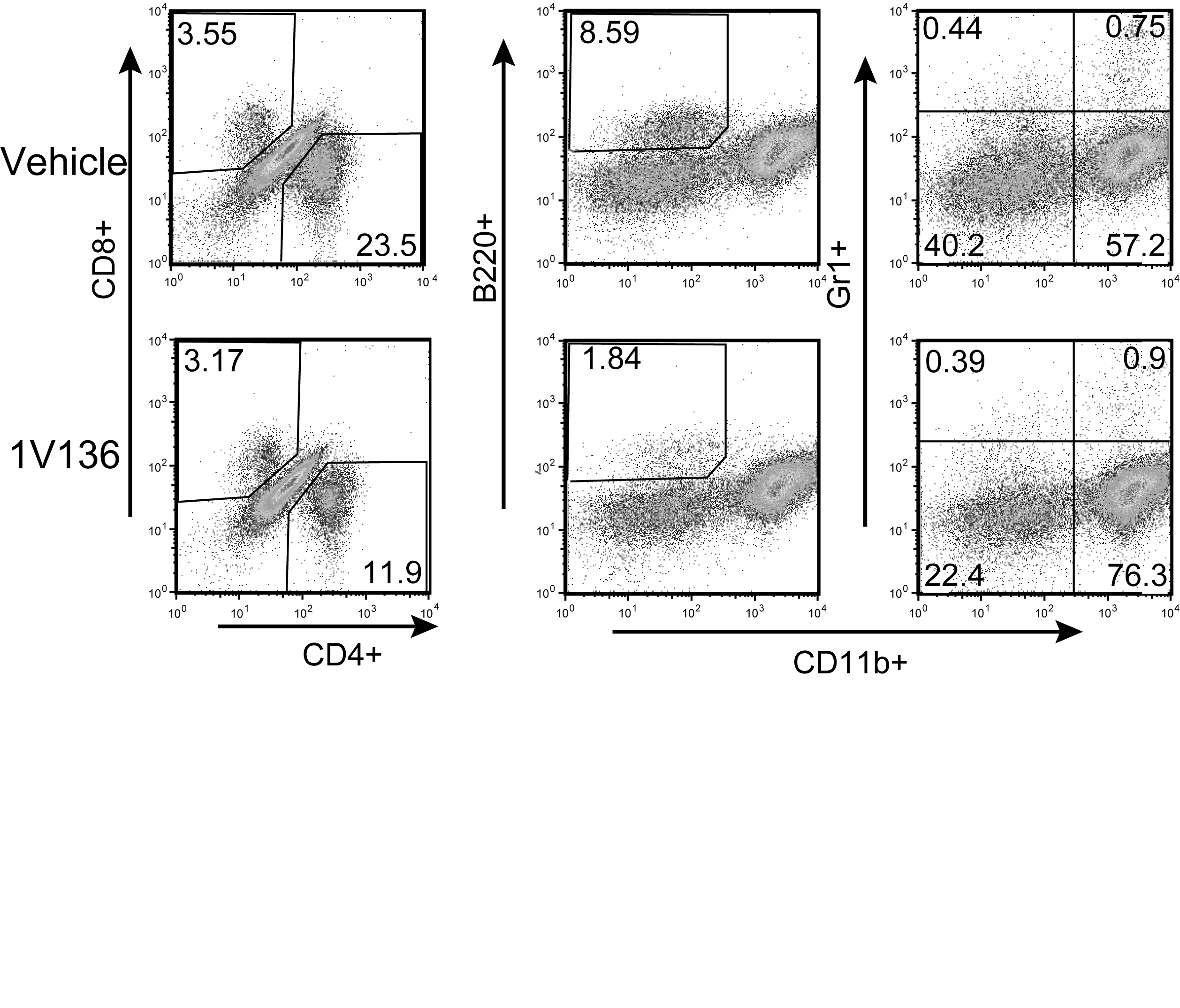

Supplement: Figure S3 — Representative FACS plots of spinal cellular infiltrates. Spinal cellular infiltrates were isolated from vehicle- (upper panels) or 1V136-treated (lower panels) EAE mice on day 19 were stained for CD4, CD8, B220, CD11b or Gr1. Data shown are representative of three independent experiments showing the similar results. (TIF) [file pone.0045860.s003.tif]

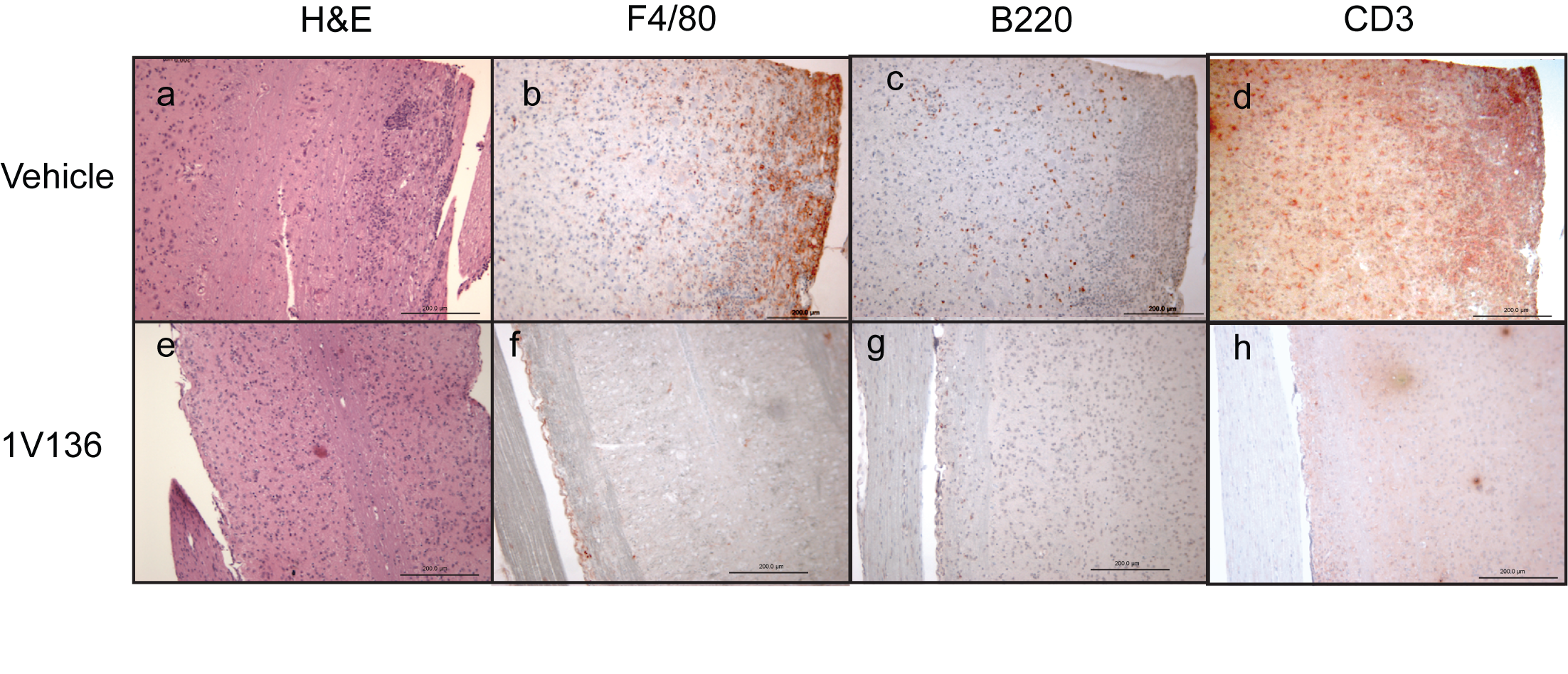

Supplement: Figure S4 — Representative inmmunohistochemistry stains of lumber spinal cords from vehicle-treated or 1V136-treated EAE mice from Figure 4 . The sections were stained H&E (a and e) and immunostained for macrophages (F4/80, b and f), B cells (B220, c and g), and T cells (CD3, d and f). × 100 original magnification. Bar indicates 200 µm. (TIF) [file pone.0045860.s004.tif]

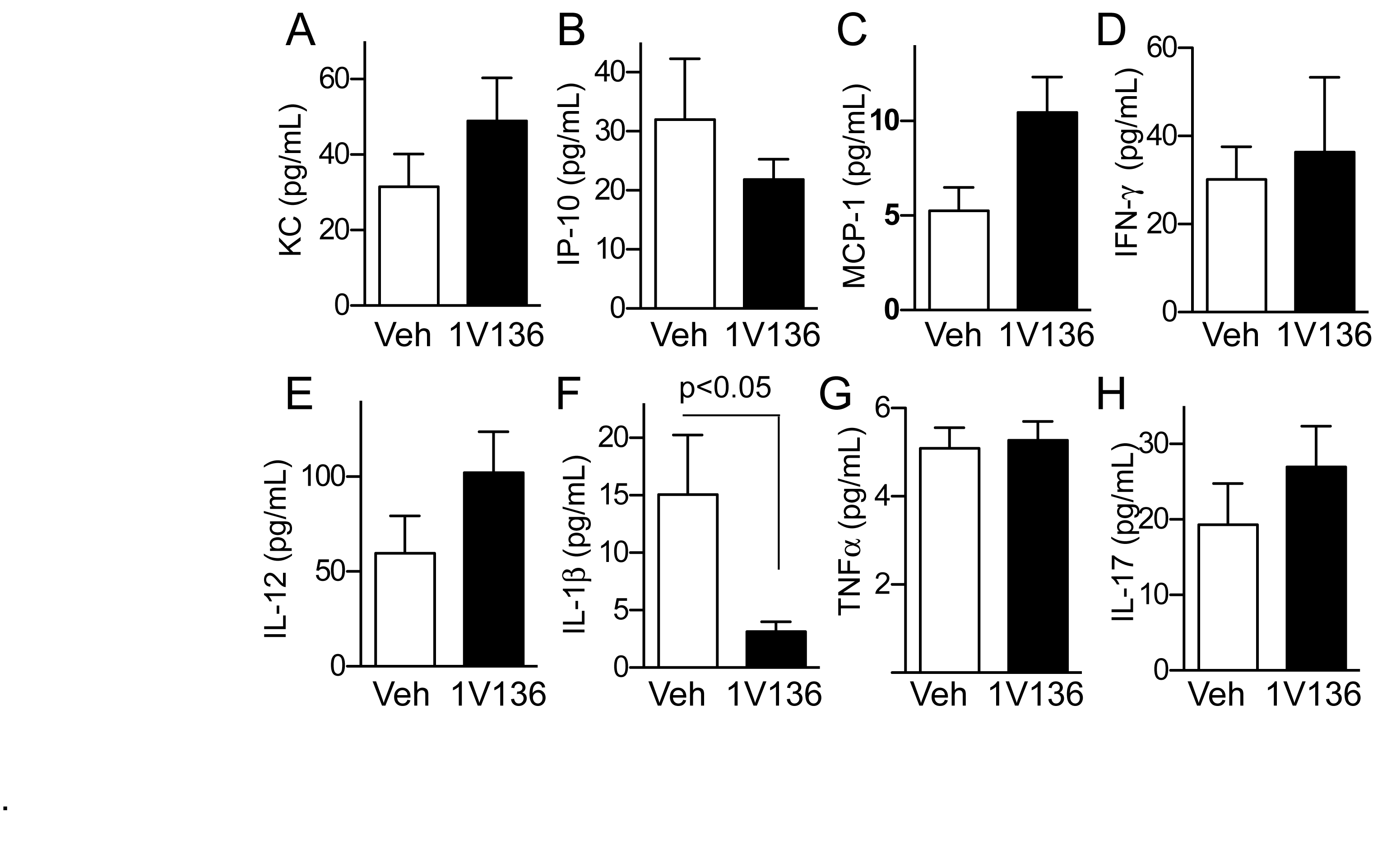

Supplement: Figure S5 — Serum cytokine profiles of PLP/EAE mice that received TLR7 ligand treatment. Sera were collected from EAE mice treated daily with vehicle (Veh) or 1V136. Levels of cytokines and chemokines were measured by Luminex beads assay. (A) KC, (B) IP-10, (C) MCP-1,(D) IFN-γ, (E) IL-12, (F) IL-1β, (G) TNFα, and (H) IL-17 levels are means ± SEM and are representative of three independent experiments. p<0.05 compared to vehicle-treated mice by Student t test. (TIF) [file pone.0045860.s005.tif]
