# Supplementary material for: Treatment of Autoimmune Inflammation by a TLR7 Ligand Regulating the Innate Immune System
Source: PLoS One. 2012 Sep 28;7(9):e45860. doi: 10.1371/journal.pone.0045860 (PMC3461028; doi:10.1371/journal.pone.0045860)
Supplement: Table S1 — Primers and probes used in this study. The sequences for the primer sets and the reference numbers for the prevalidated probes used in this study are listed. The primers were purchased from Integrated DNA Technologies, Inc. (Commercial Park. Coralville, IA) and the probes were from the Universal Probe Library (Roche Diagnostics Corporation, Indianapolis, IN). (DOCX) [file pone.0045860.s006.docx]

**Table S1.** Primers used in this study.

| **Gene** | **Left** | **Right** | **Universal Probe Library ID^1)^** |
| --- | --- | --- | --- |
| IL1b | tgtaatgaaagacggcacacc | tcttctttgggtattgcttgg | 78 |
| IP10 | gctgccgtcattttctgc | tctcactggcccgtcatc | 3 |
| KC | agactccagccacactccaa | tgacagcgcagctcattg | 83 |
| MCP-1 | catccacgtgttggctca | gatcatcttgctggtgaatgagt | 62 |
| MIP1a | caagtcttctcagcgccata | ggaatcttccggctgtagg | 40 |
| 18sRNA | aaatcagttatggttcctttggtc | gctctagaattaccacagttatccaa | 55 |

^1)^ Roche Universal Library: https://www.roche-applied-science.com/sis/rtpcr/upl

**.**
